# Can HRV Predict Prolonged Hospitalization and Favorable or Unfavorable Short-Term Outcome in Patients with Acute Ischemic Stroke?

**DOI:** 10.3390/life13040856

**Published:** 2023-03-23

**Authors:** Joanna Aftyka, Jacek Staszewski, Aleksander Dębiec, Aleksandra Pogoda-Wesołowska, Jan Żebrowski

**Affiliations:** 1 Faculty of Physics, Warsaw University of Technology, Koszykowa 75, 00-662 Warsaw, Poland; 2 Clinic of Neurology, Military Institute of Medicine, Szaserow 128, 04-141 Warsaw, Poland

**Keywords:** heart rate variability, ischemic stroke, symbolic dynamics, clinical progression, hospitalization time

## Abstract

The aim of this study was to assess whether the heart rate variability (HRV) could predict a favorable or unfavorable stroke outcome. The endpoint was based on the National Institutes of Health Stroke Scale (NIHSS). The patient’s health condition was assessed upon discharge from the hospital. An unfavorable stroke outcome was defined as death or NIHSS ≥ 9, while NIHSS < 9 meant a favorable stroke outcome. The studied group consisted of 59 patients with acute ischemic stroke AIS (mean age of 65.6 ± 13.2; 58% were females). An original and innovative non-linear measure was used to analyze HRV. It was based on symbolic dynamics consisting of comparing the “length of the longest words” in the night recording of HRV. “The length of the longest word” meant the longest sequence of identical adjacent symbols possible for a patient. An unfavorable stroke outcome occurred in 22 patients, whereas the majority of patients (37) had a favorable stroke outcome. The average hospitalization time of patients with clinical progression was 29 ± 14 days, and with favorable outcomes was 10 ± 3 days. Patients with long words (more than 150 adjacent RR intervals having the same symbol) were hospitalized no longer than 14 days and they had no clinical progression. The patients with a favorable stroke outcome were characterized by longer words. Our pilot study may be the beginning of work on the development of a non-linear, symbolic method as a predictor of prolonged hospitalization and increased risk of clinical progression in patients with AIS.

## 1. Introduction

Stroke is a sudden neurological deficit resulting from damage to the central nervous system (CNS) due to an acute vascular episode [1]. Acute ischemic stroke (AIS) is caused by the thrombotic or embolic occlusion of a cerebral artery, and it is responsible for the majority of disability-adjusted life years and is a leading cause of mortality [2]. Data from population-based observational studies estimate that incidence is high (155 cases per 100,000 inhabitants), and there will be a 13% increase in the number of first strokes in Europe by 2045 [3]. Recanalization strategies, including intravenous recombinant tissue-type plasminogen activator (rt-PA) and endovascular approaches (such as mechanical thrombectomy), reduce the consequences of stroke; however, the results depend on multiple unmodified factors such as patient age, sex, baseline ischemic core or efficacy of collateral flow. The one-month case-fatality ratio varies from 10% to 26% in various populations, and 30–50% of the survivors develop post-stroke complications (including cardiac complications, pneumonia, early recurrent strokes or secondary hemorrhages), reducing the chances of optimal recovery and thus prolonging hospitalization [4,5,6,7]. The National Institutes of Health Stroke Scale (NIHSS) is the most common method to assess stroke severity, which is directly related to further prognosis and hospitalization duration [8]. Several studies evaluating the relationship between the NIHSS or the Glasgow Coma Scale (GCS) and the length of hospital stay have been published [4,9,10,11]. Different studies on the prognosis of AIS have shown that diabetes, obesity, homocysteine and hs-CRP levels may affect the stroke-related mortality; however, predictors of stroke progression and complications in the acute phase of stroke have not been clearly elucidated. 

Heart rate variability (HRV) is one measure of autonomic nervous system (ANS) performance [12]. HRV is a record of the time series of successive RR intervals. The RR interval represents one heart cycle. It is the distance expressed in the duration between successive R waves in the electrocardiographic record. The beats originating from the sinus node are denoted as normal-to-normal (NN) intervals. HRV analysis can be performed using linear measures in the time domain, e.g., mean duration of NN intervals, linear measures in the frequency domain, which are based on the power of the spectrum in the range of specific frequencies, and non-linear measures, e.g., Sample Entropy or symbolic dynamics [13,14,15]. Several articles evaluating the relationship between stroke and HRV have been published recently [16,17,18]. This is the result of the postulates of the physiological network concept, according to which the human body is not a complex of separate systems, but an integrated network of mutually interacting systems [19]. 

In a previous report, we indicated that Sample Entropy determined from HRV records allows distinguishing patients with a right- or left-hemisphere stroke [20]. Symbolic dynamics [21] is one of the methods that was the inspiration for the original and innovative measure “the length of the longest word” proposed in this article. Moreover, there are some publications showing that HRV can be a predictor of hospitalization length for people with COVID-19 infection [22,23]. In addition, HRV measurements can also predict the prognosis of patients in the intensive care unit (ICU) on admission and on the 28th day of hospitalization, regardless of the diagnosis on admission, the treatment used and the need for mechanical ventilation [24].

Therefore, finding an indicator that would separate the group of patients at high risk of complications from these with lower risk is crucial. The group with a worse prognosis should be better monitored in order to implement appropriate actions in the early stages of clinical progression to prevent the worsening of complications and limit their negative impact on the patients’ health. Further studies are needed to define a reliable marker that will predict the length of hospitalization in patients with AIS [5,11]. This will be an important factor related to the risk of post-stroke complications and affecting the economic and administrative aspect of hospitalization, as well as important information for the family who will take care of the patient after their return home.

The aim of this study was to develop a measure that, based on the analysis of HRV in patients in the acute phase of AIS, will help to predict a favorable or unfavorable short-term ischemic stroke outcome.

## 2. Materials and Methods

### 2.1. Study Design

In this retrospective study, we analyzed the historical data of the previously described cohort with AIS admitted to the stroke unit and followed for 3 months in the outpatient department or rehabilitation department. The study protocol with detailed selection criteria and methodology has been described previously [20]. In brief, the study group consisted of 59 consecutive patients with confirmed AIS who were hospitalized at the Comprehensive Stroke Center of the Military Institute of Medicine in Warsaw between 2010 and 2019, fulfilling the pre-established eligibility criteria.

We carried out this study in accordance with the Declaration of Helsinki. The electronic database was decoded, and the patient identification data were scrambled to ensure confidentiality; informed consent was thus exempted. The evaluation of all studies, including Holter ECG monitoring or HRV interpretation, was blinded to the clinical data from the CT or MRI scans. This study was evaluated and approved as an internal study at the Military Institute of Medicine in Warsaw (no.: 00574) and by the Institutional Review Board of Military Institute of Medicine in Warsaw (no.: 20WIM/2020 at 22 April 2020).

### 2.2. Inclusion/Exclusion Criteria

The inclusion criteria consisted of (1) a 24 h Holter ECG examination performed as soon as possible following admission (earlier than 7 days after onset) of ischemic stroke; (2) a neuroimaging examination (CT on days 0 and 1, and/or MRI on day 0) to confirm AIS lesions; and (3) anterior large vessel occlusion stroke (4) reperfusion treatment (rt-PA and/or mechanical thrombectomy). The exclusion criteria for patients were: hemorrhagic or transient ischemic stroke (TIA) or unstable neurological or clinical status before Holter ECG monitoring (progressive stroke acute infections), or the presence of any ANS disorders (e.g., atypical parkinsonism). 

### 2.3. Participants

All the subjects received standard stroke diagnosis, treatment and rehabilitation according to the guidelines [25]. Each patient with atrial fibrillation received anticoagulation therapy during hospital stay; the others were given antiplatelet agents (aspirin or clopidogrel) in the secondary stroke prevention.

All patients with first-detected atrial fibrillation (AF) underwent transthoracic echocardiography to exclude valvular disease and establish the left ventricular ejection fraction (LVEF). All these patients were hemodynamically stable during hospitalization, and no patients required cardioversion. We collected data regarding the following vascular risk factors: hypertension, diabetes mellitus, dyslipidemia, currently smoking, past stroke or TIA and body mass index (BMI). We defined hypertension as systolic blood pressure ≥ 140 mm Hg or diastolic blood pressure ≥ 90 mm Hg, any use of antihypertensive drugs, or any self-reported history of hypertension. Diabetes mellitus was defined as a fasting glucose level ≥ 7.0 mmol/L, a non-fasting glucose concentration ≥ 11.1 mmol/L, any use of glucose-lowering drugs, or any self-reported history of diabetes. Dyslipidemia was defined as a serum triglyceride level ≥ 1.7 mmol/L, low-density lipoprotein cholesterol ≥ 3.6 mmol/L, high-density lipoprotein cholesterol ≤ 1.0 mmol/L, any use of lipid-lowering drugs, or any self-reported history of dyslipidemia. Coronary heart disease (CHD) was defined as a history of angina or myocardial infarction. Heart failure was diagnosed according to a classification of the European Society of Cardiology (ESC), which is based on reduced LVEF (<40%), clinical signs of heart failure, and structural and functional myocardial changes [26]. 

A total of 66 patients were screened. Only the Holter ECG records longer than 4 h were included in the study. Ultimately, 59 patients qualified for the analyses.

### 2.4. Neurological Examinations

Detailed neurological examinations were undertaken at the baseline and follow-up visits (at discharge from the hospital). Baseline and follow-up assessments were carried out by the same team of experienced investigators, who were blinded to the HRV analysis. The modified Rankin Scale (mRS) was used to evaluate functional status, and the NIHSS was used to assess neurological impairment. The patients were classified as nondependent (mRS ≤ 3 points) or dependent (mRS > 3 points). 

As the recommended duration of hospitalization according to internal standards of care in Poland is 8 days, we recognized prolonged hospitalization when it exceeded 9 days. 

### 2.5. Endpoint

The severity of stroke was categorized at discharge according to the level of disability on the NIHS scale. Unfavorable stroke outcome was defined as death or discharge NIHSS ≥ 9 (moderate or severe stroke) [27]. Due to the small size of the study group, we decided to use an overall endpoint (unfavorable/favorable outcome) based on an assessment of the neurological course of the stroke (moderate to severe deficit assessed by NIHSS) and its complications (death). However, in order to avoid subjective evaluation of the clinical condition, objective parameters such as prolonged hospitalization and death were also analyzed separately (Supplementary Materials, Tables S1 and S2). The mRS scale was not used due to the short observation time (hospital discharge). The mRS assessment has an acute floor effect (i.e., patients will receive high mRS scores because they are often bedridden while in hospital), so it is typically assessed after 3 months when patients have had the opportunity to resume their daily activities [8]. The NIHSS has a high intraobserver and interobserver reliability, reflects cerebral dysfunction by assessing several clinical items, and is responsive to meaningful clinical change. NIHSS scores range from 0 (no deficit) to 42 (maximal deficit or death) [28].

### 2.6. Heart Rate Variability

Heart rate variability changes throughout the day due to daily activity. The average heart rate accelerates as a result of physical activity and slows down during rest. In order to eliminate the impact of daily activity of a given patient, only night HRV records were analyzed. Fragments of the nocturnal ECG recordings from 10 p.m. to 6 a.m. were cut out from data from 24 h Holter ECG from 66 people. Only the records longer than 4 h were included in the study. Interruptions in the signal usually resulted from the accidental disconnection of the electrodes during sleep by the patient. Seven records were excluded for this reason. Among the remaining 59 records, occasional fragments or breaks other than normal-to-normal beats in the recording were replaced by the mean of the NN values in the sample preceding and following the ectopy, then all NN records were cut to the same number of samples as the shortest record had. The final analyses included nightly records of 59 people, including 22 patients with unfavorable stroke outcomes and 37 people with favorable stroke outcomes. The signals were recorded by the Reynolds Medical Holter ECG Lifecard CF device at a sampling rate of 128 Hz. The waves were detected automatically using Holter software. Each person in the post-stroke treatment unit had a 24 h Holter ECG examination in the acute phase of ischemic stroke (mean 5 ± 2 days following stroke onset).

### 2.7. Linear Methods 

#### 2.7.1. Time Domain

The first group of analysis methods of assessing heart rate variability were linear methods in the time domain. The following measures were compared between the group of people with unfavorable stroke outcomes and the group of people with favorable stroke outcomes: mean NN, SDNN, RMSSD, and pNN50. All measures were performed on the series of normal-to-normal intervals. Mean NN is the mean duration of NN intervals. The complementary measure of mean NN is SDNN, which indicates the deviation of the values of the NN intervals from the mean NN. Both measures are expressed in milliseconds. Another comparable indicator is RMSSD, i.e., root-mean-square successive differences between successive NN intervals. The last index is pNN50, which defines the percentage of differences in successive NN intervals greater than 50 ms in the entire signal [13]. 

#### 2.7.2. Linear Methods–Frequency Domain

The study also used a comparison of frequency analysis methods in the group of people with unfavorable or favorable stroke outcomes. The power spectra in the high-frequency (HF) and low-frequency (LF) ranges and the LF/HF ratio were compared. The HF power spectrum was analyzed in the range of 0.15–0.4 Hz, and the LF power spectrum in the range of 0.04–0.15 Hz [13].

### 2.8. Non-Linear Methods

#### 2.8.1. Sample Entropy

Sample Entropy is a measure proposed by Richman and Moorman. It was originally used as a predictor of neonatal sepsis [15]. This measure shows the complexity of the heart rhythm. In this article, it was also decided to compare the complexity of the heart rhythm recorded in the acute phase of ischemic stroke and its correlations with later complications occurring during the stay in the stroke department. When determining the Sample Entropy, r = 0.2 * standard deviation of NN intervals was assumed as the tolerance parameter, and the embedding dimension was m = 2.

#### 2.8.2. Symbolic Dynamics

Another group of non-linear methods of assessing HRV is symbolic dynamics. This method was proposed by Kurths et al. [21]. In this manuscript, as defined by Kurths et al. [21], each NN interval was replaced with the symbol {0, 1, 2, 3}. Equation (1) shows the changing of the intervals to one of the symbols:

(1) $${RR}_{n}=\left\{\begin{array}{c} \begin{array}{c} 0,\;{RR}_{n}>\left(1+a\right)\mu \\ 1,\;{RR}_{n}>\mu \&{RR}_{n}⩽\left(1+a\right)\mu \end{array}\\ \begin{array}{c} 2,\;{RR}_{n}>\left(1-a\right)\mu \&{RR}_{n}<\mu \\ 3,\;{RR}_{n}⩽\left(1-a\right)\mu \end{array} \end{array}\right.$$

μ-mean RR; a = 0.05 [21].

In our study, we only analyzed sinus node beats, i.e., normal-to-normal intervals. Arrhythmias were replaced as described in the Heart Rate Variability section. As a consequence, in our work, the RR designations in Equation (1) correspond to NN intervals instead of RR intervals.

In the original method, consecutively adjacent symbols are grouped into words of length 3, and then clustered into 0V (without variation), 1V (with 1 variation), 2LV (with 2 likely variation), and 2UV (with 2 unlikely variation) clusters. The entire signal is grouped into sequences consisting of 3 consecutive NN intervals. In the nomenclature of symbolic dynamics, the term “word” is used to describe clusters consisting of symbols, i.e., the NN intervals coded according to Equation (1). The 0V cluster contains 3-symbol words that are characterized by identical symbols, i.e., only the words {0,0,0}, {1,1,1}, {2,2,2}, and {3,3,3} can be included in this group. This means that the 0V cluster contains only words/sequences of symbols that define 3 adjacent RR intervals with the same symbol, i.e., without variation. Cluster 1V contains words with one change variation, i.e., 2 adjacent symbols are identical and 1 is different, e.g., {1,1,2}, {3,3,0}, {0,1,1}, etc. The 2V cluster contains words in which there is a double variation. The 2LV group is characterized by a two-fold likely variation, i.e., a double increase or a double decrease, e.g., {0,1,3}, {0,1,2}, {3,2,0}, etc. The 2UL group is characterized by a two-fold unlikely variation, i.e., a simultaneous decrease and increase in symbols in a word, e.g., {1,0,2}, {1,3,0}, etc.

The method proposed by us in this manuscript is our own new idea for modifying symbolic analysis. After converting each interval to one of the symbols {0,1,2,3}, it is not grouped into 3-letter words, but for each patient one word is searched for as the longest for a given subject, i.e., the longest possible sequence of identical consecutive symbols. The length (number) of the letters (symbols) in that sequence is then measured. Only one word is selected from the entire record for each patient. The longest word is characterized by identical symbols, e.g., only the symbol “3”, and there is no longer a fragment of stationarity in the entire notation, i.e., no change in symbol in a given sequence. The longest word thus determines how long the patient’s rhythm variability remains at the same level. Then, comparing favorable stroke outcome vs. unfavorable, we compared the longest (one per patient) words in both groups.

### 2.9. Statistical Analysis

For each of the analyzed variables, the Shapiro–Wilk test was performed to check the normality of the distributions. Comparisons of variables between the groups with favorable and unfavorable stroke outcome were performed using the Mann–Whitney test, because distributions did not meet the assumptions of normality. The results with *p* < 0.05 were considered statistically significant. All statistical analyses were performed in the Python 3.8 environment using built-in functions. The Bonferroni correction was used to establish the threshold of statistical significance. Additionally, using the Cohen effect size measure, the mean differences between the compared groups were determined [29]. Parameters for which Cohen’s d was 0.2 had a small effect size; 0.5, medium; and d ≥ 0.8 indicated a large effect size [30].

Correlation matrices between variables were made in the Python 3.8 environment. Pearson’s correlation was used if both variables were characterized by a normal distribution. In the remaining cases, the Spearman correlation was determined.

## 3. Results

Fifty-nine subjects were included in the analysis. The mean age of the studied cohort was 65.6 ± 13.2 years; 58% were females. The majority of patients (63%) had a favorable stroke outcome. Twenty-two patients (37% of the whole cohort) experienced an unfavorable outcome, among whom four people died. There was no significant difference in demographic or stroke risk factors between patients with a unfavorable or favorable stroke outcome (Table 1). Thirty-two patients had a discharge mRS ≥ 3 (22 fulfilled definition of unfavorable outcome, 10 had significant pre-stroke disability). Forty-one patients had prolonged hospitalization, including 18 (44%) with an unfavorable course of stroke, and 23 (56%) with a favorable outcome in whom prolonged hospitalization was due to non-stroke-related factors (the need to achieve therapeutic level of anticoagulation in AF or improvement in blood pressure control, delays in non-stroke-related diagnostic procedures, or waiting for a nursing home). The data were missing from eight patients who were transferred to other departments. 

The baseline NIHSS, as well as the discharge NIHSS and mRS and the duration of hospitalization, were significantly higher in patients with unfavorable stroke outcomes (Table 2). 

Table 3 shows the results of the analysis in the form of X (Y), where X is the mean and Y is the standard deviation. The *p*-value column represents the result of the Mann–Whitney statistical test comparing the two groups with each other, and the effect size column indicates Cohen’s d values.

In order to avoid the subjective evaluation of a favorable or unfavorable course of stroke during hospitalization, separate objective parameters such as death and prolonged hospitalization were also analyzed (Tables S1 and S2). Analyses of patients with severe infections or progressive stroke were not performed due to the low number of observations. The Supplementary Materials also include a comparison of the linear and non-linear parameters of HRV analysis depending on the mRS at discharge (≥3 or <3)—Table S3; left ventricular ejection fraction (<40% or >40%)—Table S4; use of antiarrhythmic treatment—Table S5; antiarrhythmic treatment in patients with favorable and unfavorable stroke outcomes—Tables S6 and S7.

Table 4 presents correlation coefficients between HRV analysis measures and clinical measures. A correlation coefficient equal to 1 indicates mutual correlation, −1 indicates anti-correlation, i.e., an opposite correlation, and 0 indicates a complete lack of correlation between the variables. The Supplementary Materials (Tables S8–S22) present analogous correlations, taking into account differences in sex, age and other comorbidities (hypertension, atrial fibrillation, coronary heart disease, heart failure, and diabetes). In the Supplementary Materials, Figure S1 contains a graphical representation of the correlations from Table 4.

**Figure 1:**
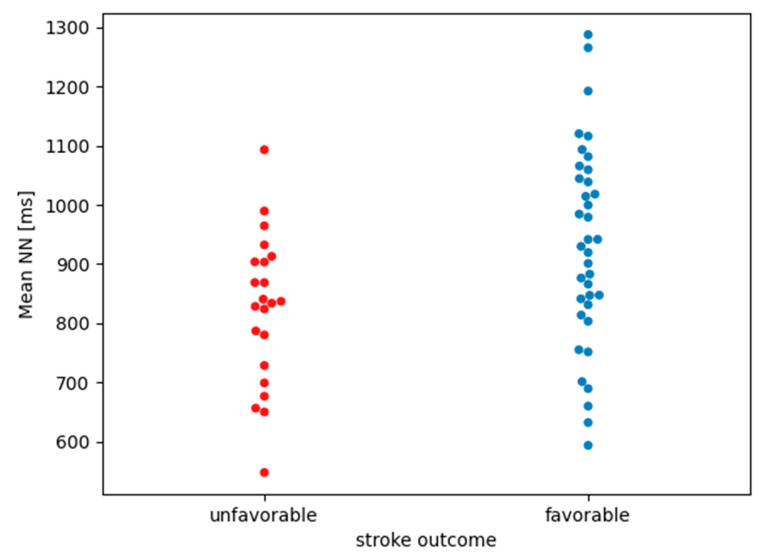
A graph comparing mean NN between groups of people with unfavorable vs. favorable stroke outcomes. Single points represent the mean NN value of a specific patient.

Among the linear parameters in the time and frequency domains and Sample Entropy (Table 3), only the mean NN comparison was statistically significant. Table 5 shows a comparison of the mean NN, standard deviations and medians in both groups. The graph (Figure 1) presents the mean NN values for each patient, divided into groups of patients with unfavorable and favorable stroke outcomes.

If the Bonferroni correction for multiple testing is used, then the statistical significance cut-off threshold changes from 0.05 to 0.05/8 = 0.006. After applying the patch, the existing difference in the mean NN of people with unfavorable vs. favorable stroke outcomes (from Table 3, extended in Table 5, presented in Figure 1) lost statistical significance (*p* = 0.015).

The symbolic dynamics methods proposed in this paper are our own modifications of the method proposed by Kurths et al. [21]. Assigning NN intervals to one of the four symbols is performed in accordance with the assumptions of Kurts et al. [21]. Then, instead of grouping the symbols into three-letter words, the longest word is made by grouping the words according to the variation number (0V 1V, 2LV, 2UV). The longest word in a signal is the longest sequence of identical symbols in the signal. A detailed description of the method can be found in the Methods chapter in the symbolic dynamics subsection. Table 6 shows the means, standard deviations and medians of the comparisons of the longest words in the groups of people with unfavorable and favorable stroke outcomes.

**Figure 2:**
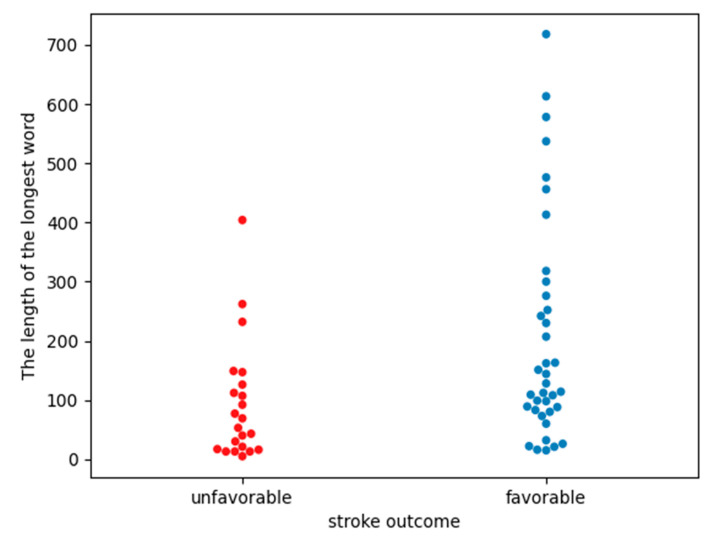
A graph comparing the lengths of the longest words between groups of people with unfavorable and favorable stroke outcomes. Single points represent the length of the longest word of a specific patient.

The Mann–Whitney statistical test (comparing the average length of the longest word between groups from Table 6) showed statistically significant differences in the length of the longest words between the groups of patients with unfavorable vs. favorable stroke outcomes (*p*-value = 0.0048). Additionally, the size of the Cohen effect was classified between medium and large as it was equal to |0.7|. In both groups, the longest words formed “3” symbols, meaning the RR intervals were shorter than 0.95 * mean NN. Figure 2 shows a comparison of the length of the longest words between the groups. The group with favorable stroke outcomes included values over the entire range of the scale, as well as in the same areas as those with unfavorable stroke outcomes.

**Figure 3:**
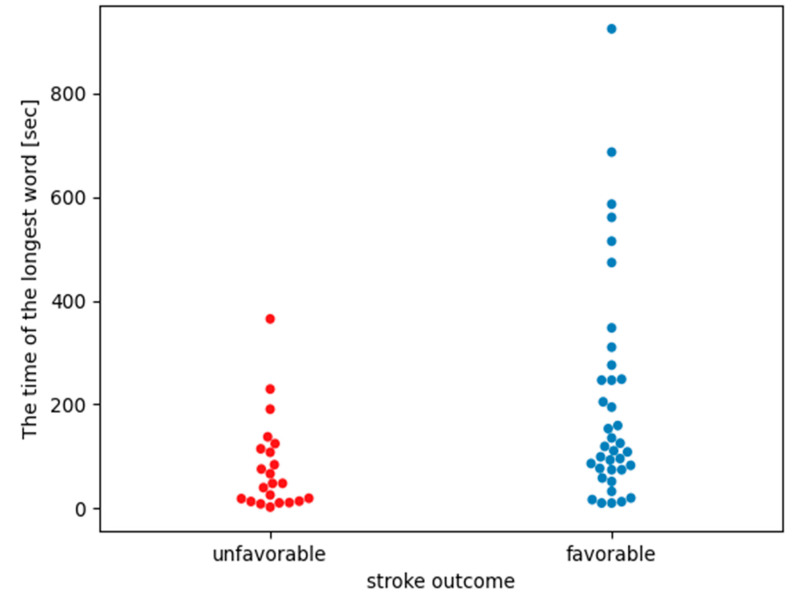
A graph comparing the time duration (in seconds) of the longest words between groups of people with unfavorable and favorable stroke outcomes. Single points represent the length of the longest word of a specific patient.

Table 7 shows the longest word durations for each patient, i.e., the number of intervals (symbols) making up the longest word for a given patient is multiplied by the mean NN for that patient. The mean, median, and standard deviation for the longest word durations are shown in Table 7, and the duration graphs for each patient are shown in Figure 3.

The results from Table 7 present the average duration of the longest word in the analyzed cohort. The Mann–Whitney statistical test, comparing the average duration of the longest word between groups from Table 7, showed statistically significant differences in the average duration of the longest words between the groups of patients with unfavorable and favorable stroke outcomes (*p*-value = 0.0042). Additionally, the size of the Cohen effect was classified between medium and large as it was equal to |0.7|. 

**Figure 4:**
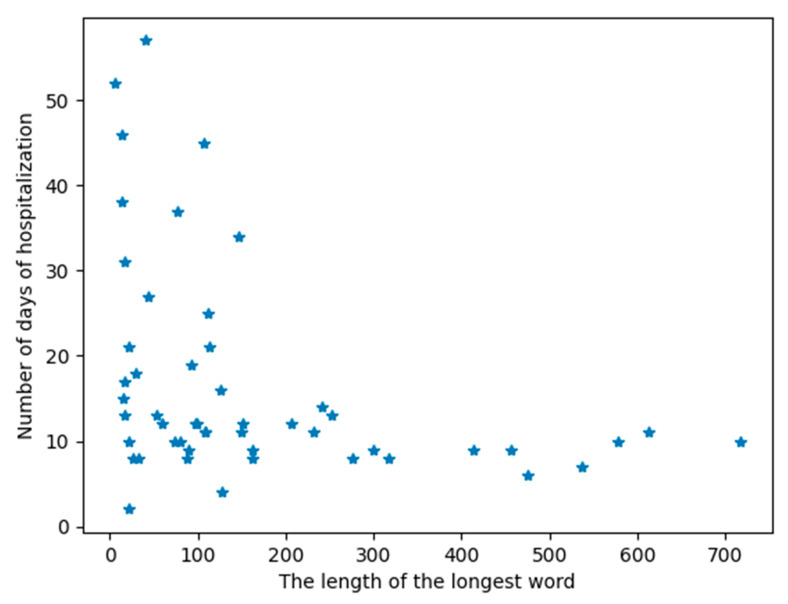
The relationship between the length of the longest word in the signal and the number of days of stay in the ward. A single point represents the data of a specific patient.

Figure 4 shows that people with the longest words (more than 150 symbols) were hospitalized for a shorter time (max. 14 days). Spearman’s correlation coefficient between the number of days of hospitalization and the length of the longest word was −0.47. The analysis of this graph shows that if a patient in the acute phase of stroke is characterized by a long word (more than 150 identical symbols adjacent to each other), then their hospitalization time will be short. If the patient has short words in the AIS, then the time of their hospitalization may be extended. It is clear that among the people who had a significantly extended hospitalization time, none of them were characterized by long words. 

## 4. Discussion

Our pilot study provides more data on the important differences in HRV measures between patients with unfavorable and favorable AIS outcomes. The results indicate that the method of symbolic dynamics may have clinical value to distinguishing patients at risk for clinical deterioration within a few days following AIS onset. However, we were unable to find a clinical deterioration predictor that would 100% separate both groups of patients from each other, because the course of stroke is influenced by many factors. We revealed that patients who had favorable stroke outcome had significantly different (longer) longest-word lengths than those with unfavorable outcomes (*p*-value = 0048). 

Surprisingly, we did not find a significant difference in the distribution of the known clinical predictors of poor stroke outcome, such as heart failure or AF. Heart failure is often characterized by signs of neurohumoral sympathetic activation, and it is considered a condition of autonomic imbalance and a risk factor for poor stroke outcome. HRV has mostly shown associations with systolic dysfunction and, more recently, with diastolic dysfunction in these patients [31]. However, in our sample we did not observe relations between heart failure and an unfavorable stroke outcome, and it only slightly correlated with the high-frequency measure (Table S4). As we did not evaluate diastolic dysfunction, which constitutes a limitation of our study, this and other echocardiography parameters will be taken into account in subsequent analyses.

Interestingly, in our cohort, among the different clinical variables, only digoxin treatment was related to an unfavorable stroke outcome (Table 1). It was reported that digoxin use was associated with an increased risk of ischemic stroke and mortality in patients with AF without heart failure, and had a neutral effect on stroke and mortality in patients with AF and heart failure [32]. The fact that digoxin could be associated with unfavorable stroke outcomes would also have wider interest than even HRV findings. However, according to the national and European guidelines, digoxin is recommended as a second-line treatment in the case of beta-blocker inefficiency, in patients with AF and LVEF < 40%, and patients on such a treatment have multimorbidity [26]. Since our group of patients receiving digoxin was small (n = 5), we conducted an analysis of the effect of all antiarrhythmic drugs (including beta-blockers), which showed no significant differences or effects on time-based HRV parameters, but demonstrated some correlations with frequency-based and non-linear parameters (LF/HF and the length of the longest word; Table S7).

The correlation analysis between linear HRV parameters in the time and frequency domains and non-linear Sample Entropy and clinical measures (Table 4) showed no strong correlations. In the Supplementary Materials (Tables S8–S22), analogous correlations are presented in relation to the sex and age of patients and the presence of comorbidities. They only indicate stronger correlations between prolonged hospitalization and short HRV parameters (mainly mean NN) in groups of patients with coronary heart disease or with heart failure. Comparing the correlation coefficient between the time of hospitalization and other measures of HRV (Table 4 and Table S8), we demonstrated that of all the HRV parameters, only the length of the longest word was most closely correlated with the time of hospitalization. The Spearman correlation coefficient between the number of days of hospitalization and the length of the longest word for a given patient was −0.47. On this basis, we can conclude that if the patient has a long word, then the hospitalization time probably will not be extended. This can also be observed in Figure 2, where the longest words appear in the group of patients with a favorable stroke outcome.

The time of hospitalization is correlated with the patient’s health condition and the occurrence of progression [9,10]. Worse patient health after a stroke and more complications are associated with longer hospitalization. The analysis of Table 2 shows that the time of hospitalization of patients with unfavorable stroke outcomes is statistically significantly different from those with favorable outcomes. As the results in Table 5 and Figure 2 show, the length of the longest word in patients with a favorable stroke outcome and those with an unfavorable outcome is significantly different. Figure 4 shows the dependence of the length of the patient’s longest word on the time of hospitalization. Table S1 in Supplementary Materials also indicates that no classical measure of HRV analysis can statistically distinguish between groups depending on the length of hospitalization. However, one should be careful in interpreting the results from Table S1, because one should not compare groups that are characterized by such a large disproportion in the number of people in each group as in Table S1.

The length of the longest word can be an indicator to distinguish a group that will require standard hospitalization (long words) from a group that may (but does not have to) have prolonged hospitalization (short words). From a medical point of view, it is better to observe a larger group, which may require longer hospitalization, whose health will deteriorate, than to miss someone with stroke complications.

In both groups, the longest words for each patient were composed of the symbol “3”, which corresponds to intervals NN < 0.95 * mean NN. Such intervals indicate that the heart rate was faster than the person’s average heart rate. During the night, we rather expect the heart rate to slow down because the body is resting and there is no daytime activity that causes changes in heart rate. The results in Table 7 and Figure 3 indicate that people in better condition had longer durations of the longest word than those with an unfavorable stroke outcome. The results presented above show that people without clinical progression had an increase in heart rate of about 2–3 min. The analysis of the tachograms (charts of heart rate variability) of these patients shows that the healthy individuals had patterns similar to the U-shapes described in Soliński et al. [33]. According to the publication [33], we can assume that a group of uncomplicated individuals had U-shaped patterns in their record. These are temporary accelerations in the night rhythm that distinguish healthy people. The group analyzed in this work had definitely longer U-shapes than in [33]. However, as Soliński et al. indicated, longer U-shapes are possible. U-shaped patterns in nighttime records are visible in the nighttime records of healthy people, and in the nighttime records of patients with cardiac diseases, these patterns disappear. If in the acute phase of a stroke it is seen that the patient has a longer word that corresponds to the pattern indicated in [33], then it can be predicted that the patient’s health condition will not deteriorate. 

The original version of symbolic dynamics analyzes only very short fragments of the record (three adjacent symbols). Our goal was to observe longer fragments of the HRV record, which are characterized by small changes in value, so we created our own modification of symbolic dynamics. According to the postulates of Soliński et al. [33], we wanted to see if patients with poorer health had fewer U-shaped behaviors than patients with better health. For this purpose, we used our own modification of symbolic dynamics. We did not use the Soliński et al. method directly, because in his work the records of healthy people and patients with cardiac diseases were analyzed. In our article, the research group was patients with stroke. Additionally, the Solinski et al. method is new and not yet fully explored, and the implementations of this method vary. We decided to check for ourselves how U-shaped behaviors can be found in the record and whether they were also visible in the group of patients with a stroke. We noticed that people with a favorable stroke outcome had longer longest words that consisted of three symbols, i.e., NN_intervals < 0.95 * mean NN. This is consistent with U-shaped patterns. This indicates that despite the stroke, people with a favorable stroke outcome have an HRV more similar to healthy people than people with an unfavorable stroke outcome due to the presence of U-shaped characteristics in the favorable stroke outcome group. Classic linear time–frequency analysis methods and Sample Entropy failed to notice this behavior because neither of these methods checks for larger stationarity/small signal variance fragments over longer NN intervals. Classical HRV linear analysis methods will not detect U-shaped patterns of behavior.

The presented work has several limitations. First, the data in our study were collected from a single center, with a small group of patients, which may limit the generalization of our results. Second, this study does not include a control group. Third, there are gaps in some patient data at 30- and 90-days post-stroke. The mRS score at 30 and 90 days were not available for all patients; therefore, we did not perform an analysis of long-term stroke results. Several independent factors that could be assessed at the time of initial presentation could be analyzed using HRV parameters in multivariate models; however, such an analysis needs a large sample of data. Therefore, our results cannot be generalized, and this study should be regarded as a pilot study. Further research is warranted.

## 5. Conclusions

The method presented in this article can be the beginning of the development of measures of symbolic dynamics that can determine patients’ complications and prolonged hospitalization after AIS on the basis of HRV recorded in the acute phase of a stroke.

## Figures and Tables

**Table 1:** Baseline characteristics of the studied group. Values are the mean ± SD for quantitative variables and n (%) for qualitative variables. The *p*-value column represents the result of the Mann–Whitney statistical test comparing the two groups with each other.

	Unfavorable Stroke Outcome	Favorable Stroke Outcome	*p*-Value
n	22 (37%)	37 (63%)	
Mean Age (SD)	65 (15)	65 (12)	0.89
Sex (f) n (%)	12 (55%)	21 (57%)	0.88
BMI (SD)	27.11 (6.46)	27.14 (4.87)	0.69
Mean NN/RR (SD)	0.99 (0.01)	0.98 (0.04)	0.29
Hemisphere (%)	L: 14 (64%);	L: 20 (54%);	L: 0.48;
	R: 5 (23%);	R: 17 (46%);	R: 0.08;
	BS: 3 (14%)	BS: 0 (0%)	BS: -
AF (%)	9 (41%)	8 (22%)	0.12
Hypertension (%)	12 (55%)	26 (70%)	0.23
Diabetes (%)	4 (18%)	6 (16%)	0.86
Smoking (%)	4 (18%)	13 (35%)	0.17
CHD (%)	4 (18%)	5 (14%)	0.64
Heart Failure (%)	5 (22%)	6 (16%)	0.55
Dyslipidemia (%)	2 (9%)	6 (16%)	0.45
Past Stroke or TIA (%)	4 (18%)	4 (11%)	0.44
Antiarrhythmic Drugs			
Digoxin (%)	4 (18%)	1 (3%)	0.04
Beta-blocker (%)	13 (59%)	21 (57%)	0.87
Others (%)	0 (0%)	2 (5%)	-

Abbreviations: BMI—body mass index; mean NN/RR—group average percentage of NN intervals among all RR intervals for a given patient; hemisphere L/R/BS—left/right/brainstem; AF—atrial fibrillation; CHD—coronary heart disease; TIA—transient ischemic attack.

**Table 2:** Course of stroke in the studied cohort. Values are the mean (SD); median (IQR) for quantitative variables and n (%) for qualitative variables. The *p*-value column represents the result of the Mann–Whitney statistical test comparing the two groups with each other.

	Unfavorable Stroke Outcome	Favorable Stroke Outcome	*p*-Value
n	22 (37%)	37 (63%)	
NIHSS baseline			0.008
mean (SD)	18.14 (5.26)	13.92 (5.90)	
median (IQR)	18.00 (16.25–20.75)	14.00 (10.00–19.00)	
NIHSS discharge			<0.001
mean (SD)	21.82 (12.26)	3.46 (2.17)	
median (IQR)	17.50 (12.25–30.25)	3.00 (2.00–5.00)	
Number of days of hospitalization			<0.001
mean (SD)	29 (14)	10 (3)	
median (IQR)	26 (17–38)	10 (8–12)	
mRS discharge			<0.001
mean (SD)	5.09 (0.54)	2.06 (1.22)	
median (IQR)	5.00 (5.00–5.00)	2.00 (1.00–3.00)	

Abbreviations: NIHSS—The National Institutes of Health Stroke Scale; mRS—modified Rankin Scale.

**Table 3:** Comparison of linear parameters in the time and frequency domains and non-linear parameters between people with unfavorable vs. favorable stroke outcomes. The values are presented in the form of X (Y), where X is the mean and Y is the standard deviation. The *p*-value column represents the result of the Mann–Whitney statistical test comparing the two groups with each other, and the effect size column indicates Cohen’s d values.

Parameter	Unfavorable Stroke Outcome (n = 22)	Favorable Stroke Outcome (n = 37)	*p*-Value	Effect Size (Cohen’s d)
Time-based analysis				
Mean NN [ms]	824 (127)	930 (169)	0.015	−0.7
SDNN [ms]	115 (63)	103 (55)	0.420	0.2
RMSSD [ms]	124 (99)	84 (75)	0.185	0.5
pNN50 [%]	39.09 (34.99)	25.46 (26.94)	0.175	0.5
Frequency-based analysis				
HF [ms^2^]	4004 (4636)	2103 (3529)	0.156	0.5
LF [ms^2^]	3176 (3019)	2247 (3520)	0.165	0.3
LF/HF	1.68 (1.63)	2.47 (2.92)	0.147	−0.3
Non-linear analysis				
Sample Entropy	1.49 (0.58)	1.38 (0.53)	0.536	0.2

**Table 4:** Comparison of correlations between HRV analysis parameters and clinical measures. The values are represented as a correlation coefficient; *p*-value. The correlation was determined using the Pearson method for normal distributions of both variables or the Spearman method when the assumption of normal distributions was not fulfilled.

	Length of Hospitalization (≤8 or >8 days)	NIHSS Discharge (≥9 or <9)	mRS Discharge (≥3 or <3)	Death	Nosocomial Pneumonia	Nosocomial Urinary Tract Infection
Mean NN	−0.16; 0.16	−0.32; 0.01	−0.36; 0.01	−0.24; 0.07	−0.29; 0.01	−0.18; 0.17
SDNN	0.04; 0.85	0.11; 0.42	0.10; 0.57	0.00; 0.98	−0.04; 0.78	−0.17; 0.20
RMSSD	0.11; 0.98	0.17; 0.19	0.28; 0.21	−0.04; 0.79	0.02; 0.89	−0.19; 0.14
pNN50	0.21; 0.45	0.18; 0.17	0.20; 0.14	−0.01; 0.94	0.02; 0.90	−0.16; 0.22
HF	0.12; 0.98	0.19; 0.16	0.31; 0.14	−0.02; 0.91	0.03; 0.83	−0.20; 0.12
LF	0.02; 0.59	0.18; 0.16	0.19; 0.21	−0.04; 0.77	−0.03; 0.81	−0.20; 0.12
LF/HF	0.13; 0.87	−0.19; 0.15	−0.18; 0.17	0.01; 0.93	−0.21; 0.11	−0.10; 0.43
Sample Entropy	0.20; 0.09	0.08; 0.54	0.26; 0.05	−0.11; 0.42	−0.05; 0.70	−0.09; 0.52

**Table 5:** Comparison of the mean, standard deviation, and median NN intervals between people with unfavorable vs. favorable stroke outcomes.

Parameter	Unfavorable Stroke Outcome (n = 22)	Favorable Stroke Outcome (n = 37)
Mean NN intervals (ms)	824	930
Median NN intervals (ms)	835	930
Standard deviation NN intervals (ms)	127	169

**Table 6:** Comparison of the mean, standard deviation, and median length of the longest words between people with unfavorable vs. favorable stroke outcomes.

Parameter	Unfavorable Stroke Outcome (n = 22)	Favorable Stroke Outcome (n = 37)
the average length of the longest word	93	206
the median length of the longest word	61	128
standard deviation of the length of the longest word	99	187

**Table 7:** Comparison of the mean, standard deviation, and median duration time of the longest words between people with unfavorable vs. favorable stroke outcomes.

Parameter	Unfavorable Stroke Outcome (n = 22)	Favorable Stroke Outcome (n = 37)
the average duration of the longest word (s)	80	206
the median duration of the longest word (s)	48	119
standard deviation of the duration of the longest word (s)	89	213

## Data Availability

The datasets used and source codes are available from the corresponding author on reasonable request.

## Supplementary Materials

The following supporting information can be downloaded at: https://www.mdpi.com/article/10.3390/life13040856/s1. Tables S1–S7 show the results of the analysis in the form of X (Y), where X is the mean and Y is the standard deviation. The p-value column represents the result of the Mann–Whitney statistical test comparing the two groups with each other, and the effect size column indicates Cohen’s d values. Tables S8–S22 present comparisons of correlations between HRV analysis parameters and clinical measurements. Figure S1: A scatter plot of the relationship between two variables. People with a favorable stroke outcome were marked in green, and people with an unfavorable stroke outcome in red. The plot was made using the pairplot function from the seaborn package (Python). The line shows the trend in the data (kind=“reg”). The X- and Y-axes contain, respectively: Mean NN, SDNN, RMSSD, pNN_50, HF, LF, LF_HF_ratio, sampEnt, NIHSS at discharge >=9 or <9, hospitalization > 8 days or <=8 days, mRS at discharge >=3 or <3, death. Correlation coefficients and *p*-values of individual pairs of variables are included in the main text in Table 4.
