# Supplementary material for: Can HRV Predict Prolonged Hospitalization and Favorable or Unfavorable Short-Term Outcome in Patients with Acute Ischemic Stroke?
Source: Life (Basel). 2023 Mar 23;13(4):856. doi: 10.3390/life13040856 (PMC10140812; doi:10.3390/life13040856)
Supplement: Supplementary file 1 [file life-13-00856-s001.zip › life-2204115-supplementary.pdf]

## Supplementary Material

Tables S1–S7 show the results of the analysis in the form of X (Y), where X is the mean and Y is the standard deviation. The p-value column represents the result of the Mann–Whitney statistical test comparing the two groups with each other, and the effect size column indicates Cohen's d values.

**Table S1.** Comparison of linear parameters in the time and frequency domains and non-linear parameters between people with length of hospitalization >8 or ≤8 days. The values are presented in the form of X (Y), where X is the mean and Y is the standard deviation. The p-value column represents the result of the Mann–Whitney statistical test comparing the two groups with each other, and the effect size column indicates Cohen's d values.

| ***Parameter*** | ***Length of hospitalization >8 days (n=41)*** | ***Length of hospitalization ≤8 days (n=10)*** | ***p-value*** | ***Effect Size (Cohen's d)*** |
|---|---|---|---|---|
| *Time-based analysis* | | | | |
| *Mean NN [ms]* | 886 (152) | 953 (209) | 0.165 | -0.4 |
| *SDNN [ms]* | 110 (62) | 104 (56) | 0.859 | 0.1 |
| *RMSSD [ms]* | 106 (95) | 82 (61) | 0.991 | 0.3 |
| *pNN50 [%]* | 33.71 (32.37) | 17.52 (20.59) | 0.455 | 0.5 |
| *Frequency-based analysis* | | | | |
| *HF [ms²]* | 3244 (4523) | 2012 (2978) | 0.991 | 0.3 |
| *LF [ms²]* | 2743 (3170) | 2539 (4886) | 0.593 | 0.1 |
| *LF/HF* | 2.43 (2.91) | 1.54 (0.90) | 0.877 | 0.3 |
| *Non-linear analysis* | | | | |
| *Sample Entropy* | 1.47 (0.53) | 1.19 (0.60) | 0.089 | 0.5 |
| *The length of the longest word* | 153 (173) | 207 (188) | 0.302 | -0.3 |

*For 8 patients from the analyzed group of 59 people, information about the length of hospitalization was missing.

**Table S2.** Comparison of linear parameters in the time and frequency domains and non-linear parameters between a group of dead vs. surviving patients. The values are presented in the form of X (Y), where X is the mean and Y is the standard deviation. The p-value column represents the result of the Mann–Whitney statistical test comparing the two groups with each other, and the effect size column indicates Cohen's d values.

| ***Parameter*** | ***Death (n=4)*** | ***Survival (n=55)*** | ***p-value*** | ***Effect Size (Cohen's d)*** |
|---|---|---|---|---|

| | | | | |
|---|---|---|---|---|
| *Time-based analysis* | | | | |
| *Mean NN [ms]* | 752 (103) | 900 (162) | 0.073 | -0.9 |
| *SDNN [ms]* | 104 (47) | 107 (59) | 0.988 | -0.1 |
| *RMSSD [ms]* | 75 (60) | 101 (88) | 0.798 | -0.3 |
| *pNN50 [%]* | 26.40 (29.99) | 30.85 (30.92) | 0.951 | -0.1 |
| *Frequency-based analysis* | | | | |
| *HF [ms$^2$]* | 1370 (1761) | 2917 (4153) | 0.916 | -0.4 |
| *LF [ms$^2$]* | 2103 (2514) | 2629 (3415) | 0.775 | -0.2 |
| *LF/HF* | 2.12 (2.15) | 2.18 (2.58) | 0.940 | -0.1 |
| *Non-linear analysis* | | | | |
| *Sample Entropy* | 1.22 (0.58) | 1.43 (0.55) | 0.424 | -0.4 |
| *The length of the longest word* | 187 (180) | 162 (169) | 0.964 | 0.1 |

**Table S3.** Comparison of linear parameters in the time and frequency domains and non-linear parameters between people with mRS at discharge ≥3 or <3. The values are presented in the form of X (Y), where X is the mean and Y is the standard deviation. The p-value column represents the result of the Mann–Whitney statistical test comparing the two groups with each other, and the effect size column indicates Cohen's d values.

| ***Parameter*** | ***mRS discharge ≥3 (n=32)*** | ***mRS discharge <3 (n=25)*** | ***p-value*** | ***Effect Size (Cohen's d)*** |
|---|---|---|---|---|
| *Time-based analysis* | | | | |
| *Mean NN [ms]* | 835 (140) | 953 (171) | 0.009 | -0.8 |
| *SDNN [ms]* | 112 (64) | 100 (51) | 0.568 | 0.2 |
| *RMSSD [ms]* | 119 (101) | 70 (55) | 0.213 | 0.6 |
| *pNN50 [%]* | 37.84 (34.56) | 20.00 (20.71) | 0.137 | 0.6 |
| *Frequency-based analysis* | | | | |
| *HF [ms$^2$]* | 3897 (4846) | 1382 (2290) | 0.141 | 0.6 |
| *LF [ms$^2$]* | 3165 (3433) | 1879 (3285) | 0.207 | 0.4 |

| | | | | |
|---|---|---|---|---|
| *LF/HF* | 2.32 (3.19) | 2.09 (1.49) | 0.174 | 0.1 |
| *Non-linear analysis* | | | | |
| *Sample Entropy* | 1.54 (0.54) | 1.26 (0.51) | 0.055 | 0.5 |
| *The length of the longest word* | 96 (94) | 255 (205) | <0.001 | 1.0 |

*For 2 patients from the analyzed group of 59 people, information about the mRS at discharge was missing.

**Table S4.** Comparison of linear parameters in the time and frequency domains and non-linear parameters between patients with left ventricular ejection fraction (EF) <40% or >40%. The values are presented in the form of X (Y), where X is the mean and Y is the standard deviation. The p-value column represents the result of the Mann–Whitney statistical test comparing the two groups with each other, and the effect size column indicates Cohen's d values.

| ***Parameter*** | ***EF < 40% (n=7)*** | ***EF > 40%(n=43)*** | ***p-value*** | ***Effect Size (Cohen's d)*** |
|---|---|---|---|---|
| *Time-based analysis* | | | | |
| *Mean NN [ms]* | 886 (90) | 909 (169) | 0.675 | -0.1 |
| *SDNN [ms]* | 142 (70) | 109 (56) | 0.252 | 0.6 |
| *RMSSD [ms]* | 153 (89) | 100 (88) | 0.061 | 0.6 |
| *pNN50 [%]* | 40.57 (29.76) | 32.50 (31.50) | 0.314 | 0.3 |
| *Frequency-based analysis* | | | | |
| *HF [ms$^2$]* | 5084 (4698) | 2854 (4175) | 0.044 | 0.5 |
| *LF [ms$^2$]* | 5233 (5533) | 2561 (3006) | 0.069 | 0.8 |
| *LF/HF* | 1.08 (0.49) | 2.57 (2.86) | 0.219 | -0.6 |
| *Non-linear analysis* | | | | |
| *Sample Entropy* | 1.18 (0.56) | 1.55 (0.51) | 0.083 | -0.7 |
| *The length of the longest word* | 104 (51) | 167 (174) | 0.665 | -0.4 |

*For 9 patients from the analyzed group of 59 people, information about the left ventricular EF% was missing.

**Table S5.** Comparison of linear parameters in the time and frequency domains and non-linear parameters between patients taking antiarrhythmic medicament vs. without antiarrhythmic medicament. The values are presented in the form of X (Y), where X is the mean and Y is the standard deviation. The p-value column represents the result

of the Mann–Whitney statistical test comparing the two groups with each other, and the effect size column indicates Cohen's d values.

| *Parameter* | *Antiarrhythmic medicament (n=35)* | *Without antiarrhythmic medicament (n=24)* | *p-value* | *Effect Size (Cohen's d)* |
|---|---|---|---|---|
| *Time-based analysis* | | | | |
| *Mean NN [ms]* | 868 (167) | 922 (152) | 0.149 | -0.3 |
| *SDNN [ms]* | 115 (62) | 96 (50) | 0.320 | 0.3 |
| *RMSSD [ms]* | 123 (94) | 65 (60) | 0.037 | 0.7 |
| *pNN50 [%]* | 37.35 (33.15) | 20.62 (23.83) | 0.060 | 0.6 |
| *Frequency-based analysis* | | | | |
| *HF [ms²]* | 3790 (4467) | 1386 (2864) | 0.041 | 0.6 |
| *LF [ms²]* | 3199 (3853) | 1711 (2220) | 0.214 | 0.5 |
| *LF/HF* | 1.43 (1.30) | 3.26 (3.41) | <0.001 | -0.8 |
| *Non-linear analysis* | | | | |
| *Sample Entropy* | 1.50 (0.59) | 1.31 (0.47) | 0.214 | 0.4 |
| *The length of the longest word* | 124 (146) | 221 (185) | 0.016 | -0.6 |

**Table S6.** Comparison of linear parameters in the time and frequency domains and non-linear parameters between patients taking antiarrhythmic medicament vs. without antiarrhythmic medicament in the group of people with a favorable stroke outcome. The values are presented in the form of X (Y), where X is the mean and Y is the standard deviation. The p-value column represents the result of the Mann–Whitney statistical test comparing the two groups with each other, and the effect size column indicates Cohen's d values.

| *Parameter* | *Antiarrhythmic medicament -favorable stroke outcome (n=13)* | *Without antiarrhythmic medicament - favorable stroke outcome (n=9)* | *p-value* | *Effect Size (Cohen's d)* |
|---|---|---|---|---|
| *Time-based analysis* | | | | |
| *Mean NN [ms]* | 816 (128) | 835 (133) | 0.947 | -0.1 |
| *SDNN [ms]* | 129 (59) | 95 (66) | 0.285 | 0.5 |

| | | | | |
|---|---|---|---|---|
| *RMSSD [ms]* | 148 (102) | 89 (90) | 0.205 | 0.6 |
| *pNN50 [%]* | 46.47 (36.06) | 28.43 (32.37) | 0.171 | 0.5 |
| *Frequency-based analysis* | | | | |
| *HF [ms²]* | 4916 (4724) | 2688 (4431) | 0.317 | 0.5 |
| *LF [ms²]* | 3416 (2988) | 2831 (3210) | 0.548 | 0.2 |
| *LF/HF* | 1.09 (1.08) | 2.52 (1.96) | 0.013 | -1.0 |
| *Non-linear analysis* | | | | |
| *Sample Entropy* | 1.53 (0.70) | 1.42 (0.37) | 0.640 | 0.2 |
| *The length of the longest word* | 99 (116) | 84 (75) | 0.688 | 0.1 |

**Table S7.** Comparison of linear parameters in the time and frequency domains and non-linear parameters between patients taking antiarrhythmic medicament vs. without antiarrhythmic medicament in the group of people with an unfavorable stroke outcome. The values are presented in the form of X (Y), where X is the mean and Y is the standard deviation. The p-value column represents the result of the Mann–Whitney statistical test comparing the two groups with each other, and the effect size column indicates Cohen's d values.

| ***Parameter*** | ***Antiarrhythmic medicament -unfavorable stroke outcome (n=22)*** | ***Without antiarrhythmic medicament - unfavorable stroke outcome (n=15)*** | ***p-value*** | ***Effect Size (Cohen's d)*** |
|---|---|---|---|---|
| *Time-based analysis* | | | | |
| *Mean NN [ms]* | 898 (183) | 975 (142) | 0.118 | -0.5 |
| *SDNN [ms]* | 107 (64) | 96 (39) | 0.988 | 0.2 |
| *RMSSD [ms]* | 108 (88) | 50 (27) | 0.111 | 0.8 |
| *pNN50 [%]* | 31.96 (30.89) | 15.94 (16.48) | 0.169 | 0.6 |
| *Frequency-based analysis* | | | | |
| *HF [ms²]* | 3124 (4279) | 605 (715) | 0.092 | 0.8 |
| *LF [ms²]* | 3071 (4345) | 1039 (959) | 0.330 | 0.6 |
| *LF/HF* | 1.63 (1.39) | 3.71 (4.04) | 0.021 | -0.7 |

| Non-linear analysis | | | | |
|---|---|---|---|---|
| *Sample Entropy* | 1.49 (0.53) | 1.24 (0.52) | 0.178 | 0.5 |
| *The length of the longest word* | 139 (161) | 304 (184) | 0.001 | -1.0 |

**Table S8.** Comparison of correlations between HRV analysis parameters and clinical measures. The values are represented as a correlation coefficient; p-value. The correlation was determined using the Pearson method for normal distributions of both variables or the Spearman method when the assumption of normal distributions was not fulfilled.

| | *Length of hospitalization* | *NIHSS discharge* | *mRS discharge* | *Death* | *Nosocomial pneumonia* | *Nosocomial urinary tract infection* |
|---|---|---|---|---|---|---|
| *Mean NN* | -0.39; 0.004 | -0.40; 0.002 | -0.41; 0.001 | -0.24; 0.07 | -0.29; 0.01 | -0.18; 0.17 |
| *SDNN* | -0.05; 0.99 | 0.01; 0.91 | 0.05; 0.68 | 0.00; 0.98 | -0.04; 0.78 | -0.17; 0.20 |
| *RMSSD* | 0.15; 0.60 | 0.04; 0.78 | 0.21; 0.39 | -0.04; 0.79 | 0.02; 0.89 | -0.19; 0.14 |
| *pNN50* | 0.14; 0.31 | 0.06; 0.64 | 0.17; 0.21 | -0.01; 0.94 | 0.02; 0.90 | -0.16; 0.22 |
| *HF* | 0.19; 0.52 | 0.06; 0.67 | 0.22; 0.29 | -0.02; 0.91 | 0.03; 0.83 | -0.20; 0.12 |
| *LF* | 0.05; 0.53 | 0.07; 0.60 | 0.08; 0.34 | -0.04; 0.77 | -0.03; 0.81 | -0.20; 0.12 |
| *LF/HF* | -0.21; 0.14 | -0.08; 0.53 | -0.17; 0.20 | 0.01; 0.93 | -0.21; 0.11 | -0.10; 0.43 |
| *Sample Entropy* | 0.31; 0.03 | 0.04; 0.79 | 0.21; 0.12 | -0.11; 0.42 | -0.05; 0.70 | -0.09; 0.52 |

**Table S9.** Comparison of correlations between HRV analysis parameters and clinical measures in a group of men. The values are represented as a correlation coefficient; p-value. The correlation was determined using the Pearson method for normal distributions of both variables or the Spearman method when the assumption of normal distributions was not fulfilled.

| | *Length of hospitalization (≤8 or >8 days)* | *NIHSS discharge (≥9 or <9)* | *mRS discharge (≥3 or <3)* | *Death* | *Nosocomial pneumonia* | *Nosocomial urinary tract infection* |
|---|---|---|---|---|---|---|
| *Mean NN* | -0.27; | -0.38; | -0.51; | -0.47; | -0.46; | 0.10; |

| | | | | | | |
|---|---|---|---|---|---|---|
| | <0.001 | 0.001 | 0.004 | <0.001 | <0.001 | 0.23 |
| *SDNN* | -0.21; 0.02 | 0.13; 0.62 | 0.11; 0.93 | -0.06; 0.42 | -0.19; 0.25 | -0.08; 0.26 |
| *RMSSD* | -0.04; 0.25 | 0.19; 0.70 | 0.33; 0.39 | -0.04; 0.96 | -0.09; 0.86 | -0.21; 0.03 |
| *pNN50* | 0.10; 0.30 | 0.17; 0.79 | 0.26; 0.37 | 0.02; 0.94 | -0.03; 0.82 | -0.17; 0.06 |
| *HF* | -0.06; 0.24 | 0.27; 0.60 | 0.28; 0.39 | 0.04; 0.89 | -0.01; 0.97 | -0.23; 0.02 |
| *LF* | -0.33; 0.03 | 0.20; 0.96 | 0.16; 0.85 | 0.00; 0.59 | -0.14; 0.40 | -0.17; 0.11 |
| *LF/HF* | 0.10; 0.69 | -0.22; 0.39 | -0.15; 0.25 | -0.07; 0.47 | -0.21; 0.46 | 0.10; 0.02 |
| *Sample Entropy* | 0.37; 0.10 | 0.14; 0.38 | 0.48 | 0.12; 0.22 | 0.18; 0.19 | -0.04; 0.29 |

**Table S10.** Comparison of correlations between HRV analysis parameters and clinical measures in a group of women. The values are represented as a correlation coefficient; p-value. The correlation was determined using the Pearson method for normal distributions of both variables or the Spearman method when the assumption of normal distributions was not fulfilled.

| | *Length of hospitalization (≤8 or >8 days)* | *NIHSS discharge (≥9 or <9)* | *mRS discharge (≥3 or <3)* | *Death* | *Nosocomial pneumonia* | *Nosocomial urinary tract infection* |
|---|---|---|---|---|---|---|
| *Mean NN* | -0.19; <0.001 | -0.26; <0.001 | -0.25; 0.007 | 0.07; 0.68 | -0.14; 0.005 | -0.39; <0.001 |
| *SDNN* | 0.12; 0.35 | 0.07; 0.25 | 0.09; 0.58 | 0.02; 0.06 | 0.08; 0.28 | -0.24; 0.13 |
| *RMSSD* | 0.18; 0.42 | 0.14; 0.55 | 0.24; 0.87 | -0.09; 0.02 | 0.09; 0.58 | -0.19; 0.23 |
| *pNN50* | 0.27; 0.55 | 0.17; 0.62 | 0.16; 0.82 | -0.05; 0.04 | 0.04; 0.46 | -0.15; 0.29 |
| *HF* | 0.21; 0.68 | 0.11; 0.77 | 0.28; 0.62 | -0.11; 0.03 | 0.08; 0.76 | -0.20; 0.38 |
| *LF* | 0.25; 0.28 | 0.13; 0.33 | 0.28; 0.79 | -0.17; 0.01 | 0.05; 0.33 | -0.24; 0.12 |
| *LF/HF* | 0.07; 0.63 | -0.17; 0.91 | -0.19; 0.92 | 0.09; 0.03 | -0.25; 0.92 | -0.37; 0.86 |

| | | | | | | |
|---|---|---|---|---|---|---|
| *Sample Entropy* | 0.25; 1.00 | 0.07; 0.42 | 0.10; 0.98 | -0.26; 0.002 | -0.17; 0.19 | -0.08; 0.56 |

**Table S11.** Comparison of correlations between HRV analysis parameters and clinical measures in the group of patients aged 65 and over. The values are represented as a correlation coefficient; p-value. The correlation was determined using the Pearson method for normal distributions of both variables or the Spearman method when the assumption of normal distributions was not fulfilled.

| | *Length of hospitalization (≤8 or >8 days)* | *NIHSS discharge (≥9 or <9)* | *mRS discharge (≥3 or <3)* | *Death* | *Nosocomial pneumonia* | *Nosocomial urinary tract infection* |
|---|---|---|---|---|---|---|
| *Mean NN* | -0.12; 0.003 | -0.15; 0.01 | -0.17; 0.37 | -0.11; 0.32 | -0.23; 0.01 | -0.41; <0.001 |
| *SDNN* | 0.03; 0.24 | 0.21; 0.30 | 0.21; 0.39 | -0.15; 0.005 | -0.04; 0.12 | -0.26; 0.07 |
| *RMSSD* | 0.14; 0.50 | 0.20; 0.58 | 0.25; 0.13 | -0.24; 0.004 | 0.01; 0.36 | -0.23; 0.37 |
| *pNN50* | 0.22; 0.72 | 0.22; 0.74 | 0.31; 0.08 | -0.21; 0.004 | 0.01; 0.46 | -0.19; 0.49 |
| *HF* | 0.14; 0.49 | 0.20; 0.58 | 0.32; 0.11 | -0.20; 0.007 | 0.00; 0.37 | -0.25; 0.31 |
| *LF* | -0.04; 0.22 | 0.15; 0.21 | 0.15; 0.48 | -0.27; 0.002 | -0.09; 0.08 | -0.28; 0.05 |
| *LF/HF* | 0.07; 0.52 | -0.24; 0.81 | -0.35; 0.10 | 0.17; 0.04 | -0.16; 0.89 | -0.30; 0.35 |
| *Sample Entropy* | 0.17; 0.86 | 0.00; 0.62 | 0.22; 0.11 | -0.27; 0.001 | -0.22; 0.31 | -0.04; 0.89 |

**Table S12.** Comparison of correlations between HRV analysis parameters and clinical measures in the group of patients under 65 years of age. The values are represented as a correlation coefficient; p-value. The correlation was determined using the Pearson method for normal distributions of both variables or the Spearman method when the assumption of normal distributions was not fulfilled.

| | *Length of hospitalization (≤8 or >8 days)* | *NIHSS discharge (≥9 or <9)* | *mRS discharge (≥3 or <3)* | *Death* | *Nosocomial pneumonia* | *Nosocomial urinary tract infection* |
|---|---|---|---|---|---|---|
| *Mean NN* | -0.26; <0.001 | -0.51; <0.001 | -0.54; <0.001 | -0.40; 0.03 | -0.38; <0.001 | 0.13; 0.21 |
| *SDNN* | 0.07; 0.01 | 0.05; 0.23 | -0.09; 0.08 | 0.20; 0.82 | -0.03; 0.35 | -0.02; 0.73 |

| | | | | | | |
|---|---|---|---|---|---|---|
| *RMSSD* | 0.12; 0.04 | 0.18; 0.59 | 0.03; 0.30 | 0.24; 0.94 | 0.06; 0.74 | -0.16; 0.15 |
| *pNN50* | 0.25; 0.10 | 0.15; 0.62 | 0.04; 0.35 | 0.25; 0.94 | 0.00; 0.67 | -0.13; 0.24 |
| *HF* | 0.15; 0.06 | 0.21; 0.82 | 0.07; 0.46 | 0.27; 0.58 | 0.08; 0.98 | -0.16; 0.13 |
| *LF* | 0.22; 0.09 | 0.19; 0.56 | 0.11; 0.32 | 0.27; 0.75 | 0.03; 0.58 | -0.11; 0.48 |
| *LF/HF* | 0.18; 0.25 | -0.18; 0.66 | 0.04; 0.99 | -0.02; 0.48 | -0.16; 0.54 | 0.18; 0.02 |
| *Sample Entropy* | 0.30; 0.22 | 0.22; 0.09 | 0.30; 0.11 | 0.05; 0.39 | 0.08; 0.19 | -0.15; 0.04 |

**Table S13.** Comparison of correlations between HRV analysis parameters and clinical measures in a group of patients with hypertension. The values are represented as a correlation coefficient; p-value. The correlation was determined using the Pearson method for normal distributions of both variables or the Spearman method when the assumption of normal distributions was not fulfilled.

| | *Length of hospitalization (≤8 or >8 days)* | *NIHSS discharge (≥9 or <9)* | *mRS discharge (≥3 or <3)* | *Death* | *Nosocomial pneumonia* | *Nosocomial urinary tract infection* |
|---|---|---|---|---|---|---|
| *Mean NN* | 0.02; 0.01 | -0.15; 0.05 | -0.30; <0.001 | -0.12; 0.12 | -0.22; 0.04 | -0.21; 0.008 |
| *SDNN* | -0.10; 0.01 | 0.22; 0.97 | 0.04; 0.57 | 0.09; 0.89 | 0.08; 0.83 | -0.20; 0.003 |
| *RMSSD* | 0.01; 0.04 | 0.27; 0.46 | 0.14; 0.68 | 0.05; 0.61 | 0.16; 0.59 | -0.23; 0.01 |
| *pNN50* | 0.11; 0.06 | 0.30; 0.34 | 0.17; 0.56 | 0.08; 0.44 | 0.17; 0.46 | -0.19; 0.03 |
| *HF* | 0.00; 0.03 | 0.24; 0.39 | 0.11; 0.60 | 0.05; 0.48 | 0.14; 0.50 | -0.24; 0.02 |
| *LF* | -0.14; 0.01 | 0.21; 0.97 | 0.08; 0.78 | -0.03; 0.94 | 0.01; 0.77 | -0.24; <0.001 |
| *LF/HF* | 0.14; 0.45 | -0.28; 0.11 | -0.23; 0.12 | -0.15; 0.24 | -0.35; 0.14 | -0.15; 0.35 |
| *Sample Entropy* | 0.21; 0.65 | 0.15; 0.37 | 0.35; 0.09 | -0.03; 0.53 | 0.04; 0.48 | -0.07; 0.27 |

**Table S14.** Comparison of correlations between HRV analysis parameters and clinical measures in a group of patients without hypertension. The values are represented as a correlation coefficient; p-value. The correlation was determined using the Pearson method for normal distributions of both variables or the Spearman method when the assumption of normal distributions was not fulfilled.

| | *Length of hospitalization (≤8 or >8 days)* | *NIHSS discharge (≥9 or <9)* | *mRS discharge (≥3 or <3)* | *Death* | *Nosocomial pneumonia* | *Nosocomial urinary tract infection* |
|---|---|---|---|---|---|---|
| *Mean NN* | -0.50; 0.03 | -0.65; <0.001 | -0.49; 0.002 | -0.46; <0.001 | -0.46; <0.001 | - |
| *SDNN* | 0.31; 0.74 | -0.06; 0.07 | 0.21; 0.59 | -0.05; 0.06 | -0.18; 0.02 | - |
| *RMSSD* | 0.31; 0.71 | 0.02; 0.25 | 0.37; 0.87 | -0.19; 0.05 | -0.22; 0.05 | - |
| *pNN50* | 0.43; 0.71 | -0.08; 0.11 | 0.23; 0.79 | -0.19; 0.04 | -0.28; 0.02 | - |
| *HF* | 0.33; 0.64 | 0.08; 0.29 | 0.38; 0.80 | -0.19; 0.05 | -0.17; 0.06 | - |
| *LF* | 0.34; 0.30 | 0.11; 0.49 | 0.43; 0.59 | -0.02; 0.12 | -0.11; 0.14 | - |
| *LF/HF* | 0.08; 0.66 | 0.03; 0.25 | 0.14; 0.86 | 0.24; 0.04 | 0.13; 0.05 | - |
| *Sample Entropy* | 0.23; 0.99 | -0.13; 0.05 | 0.07; 0.63 | -0.29; 0.005 | -0.28; 0.006 | - |

*Too small a group of patients with arterial hypertension and nosocomial urinary tract infection to make a correlation.

**Table S15.** Comparison of correlations between HRV analysis parameters and clinical measures in a group of patients with atrial fibrillation. The values are represented as a correlation coefficient; p-value. The correlation was determined using the Pearson method for normal distributions of both variables or the Spearman method when the assumption of normal distributions was not fulfilled.

| | *Length of hospitalization (≤8 or >8 days)* | *NIHSS discharge (≥9 or <9)* | *mRS discharge (≥3 or <3)* | *Death* | *Nosocomial pneumonia* | *Nosocomial urinary tract infection* |
|---|---|---|---|---|---|---|
| *Mean NN* | -0.77; 0.25 | -0.22; 0.28 | -0.38; 0.61 | -0.31; <0.001 | 0.03; 0.85 | -0.41; 0.002 |
| *SDNN* | 0.52; 0.005 | 0.41; 0.06 | 0.70; 0.002 | -0.26; 0.35 | 0.24; 0.24 | -0.34; 0.01 |
| *RMSSD* | 0.64; 0.005 | 0.41; 0.07 | 0.78; 0.002 | -0.26; 0.35 | 0.34; 0.18 | -0.30; 0.02 |
| *pNN50* | 0.74; 0.003 | 0.29; 0.12 | 0.66; 0.001 | -0.26; 0.39 | 0.24; 0.31 | -0.30; 0.05 |

| | | | | | | |
|---|---|---|---|---|---|---|
| *HF* | 0.59; 0.005 | 0.38; 0.08 | 0.84; 0.005 | -0.26; 0.35 | 0.26; 0.20 | -0.37; 0.01 |
| *LF* | 0.50; 0.005 | 0.38; 0.08 | 0.68; 0.13 | -0.26; 0.35 | 0.26; 0.20 | -0.37; 0.01 |
| *LF/HF* | -0.81; 0.06 | -0.36; 0.03 | 0.59; 0.09 | -0.36; 0.002 | -0.21; 0.11 | 0.04; 0.25 |
| *Sample Entropy* | 0.58; 0.05 | 0.17; 0.25 | 0.34; 0.58 | 0.05; 0.05 | -0.11; 0.89 | 0.04; 0.07 |

**Table S16.** Comparison of correlations between HRV analysis parameters and clinical measures in a group of patients without atrial fibrillation. The values are represented as a correlation coefficient; p-value. The correlation was determined using the Pearson method for normal distributions of both variables or the Spearman method when the assumption of normal distributions was not fulfilled.

| | *Length of hospitalization (≤8 or >8 days)* | *NIHSS discharge (≥9 or <9)* | *mRS discharge (≥3 or <3)* | *Death* | *Nosocomial pneumonia* | *Nosocomial urinary tract infection* |
|---|---|---|---|---|---|---|
| *Mean NN* | 0.07; 0.01 | -0.35; <0.001 | -0.31; <0.001 | -0.24; 0.03 | -0.43; <0.001 | -0.09; 0.02 |
| *SDNN* | -0.21; <0.001 | -0.19; 0.002 | -0.29; <0.001 | 0.08; 0.25 | -0.27; <0.001 | -0.15; 0.004 |
| *RMSSD* | -0.31; <0.001 | -0.13; 0.03 | -0.20; 0.01 | -0.03; 0.28 | -0.22; 0.06 | -0.23; <0.001 |
| *pNN50* | -0.08; 0.001 | -0.14; 0.01 | -0.15; 0.01 | 0.00; 0.19 | -0.23; 0.02 | -0.19; <0.001 |
| *HF* | -0.40; <0.001 | -0.09; 0.05 | -0.17; 0.01 | 0.01; 0.42 | -0.19; 0.10 | -0.25; <0.001 |
| *LF* | -0.26; 0.001 | -0.07; 0.01 | -0.11; 0.002 | -0.02; 0.26 | -0.28; 0.001 | -0.20; <0.001 |
| *LF/HF* | 0.27; 0.24 | -0.04; 0.50 | 0.02; 0.78 | 0.05; 0.71 | -0.22; 0.20 | -0.03; 0.30 |
| *Sample Entropy* | 0.05; 0.91 | -0.18; 0.13 | 0.03; 0.82 | -0.19; 0.08 | -0.21; 0.16 | -0.19; 0.23 |

**Table S17.** Comparison of correlations between HRV analysis parameters and clinical measures in a group of patients with coronary heart disease. The values are represented as a correlation coefficient; p-value. The correlation was determined using the Pearson method for normal distributions of both variables or the Spearman method when the assumption of normal distributions was not fulfilled.

| | *Length of hospitalization (≤8 or >8 days)* | *NIHSS discharge (≥9 or <9)* | *mRS discharge (≥3 or <3)* | *Death* | *Nosocomial pneumonia* | *Nosocomial urinary tract infection* |
|---|---|---|---|---|---|---|
| *Mean NN* | -0.83; <0.001 | 0.01; 0.84 | 0.00; 0.38 | - | 0.09; 0.38 | -0.55; 0.06 |
| *SDNN* | -0.57; 0.07 | 0.19; 0.16 | 0.00; 0.18 | - | 0.09; 0.94 | -0.55; <0.001 |
| *RMSSD* | -0.18; 0.53 | 0.29; 0.02 | 0.00; 0.01 | - | 0.17; 0.41 | -0.55; <0.001 |
| *pNN50* | 0.02; 0.53 | 0.35; 0.07 | 0.65; 0.03 | - | 0.35; 0.28 | -0.55; <0.001 |
| *HF* | -0.23; 0.53 | 0.23; 0.02 | 0.00; 0.01 | - | 0.09; 0.45 | -0.55; <0.001 |
| *LF* | -0.85; 0.07 | -0.03; 0.16 | 0.00; 0.18 | - | 0.09; 0.94 | -0.55; <0.001 |
| *LF/HF* | -0.68; 0.003 | -0.13; 0.11 | 0.00; 0.01 | - | -0.35; 0.04 | 0.14; 0.89 |
| *Sample Entropy* | 0.39; 0.05 | 0.11; 0.37 | 0.00; 0.08 | - | 0.43; 0.009 | 0.00; 0.69 |

*Too small a group of patients with coronary heart disease and death to make a correlation.

**Table S18.** Comparison of correlations between HRV analysis parameters and clinical measures in a group of patients without coronary heart disease. The values are represented as a correlation coefficient; p-value. The correlation was determined using the Pearson method for normal distributions of both variables or the Spearman method when the assumption of normal distributions was not fulfilled.

| | *Length of hospitalization (≤8 or >8 days)* | *NIHSS discharge (≥9 or <9)* | *mRS discharge (≥3 or <3)* | *Death* | *Nosocomial pneumonia* | *Nosocomial urinary tract infection* |
|---|---|---|---|---|---|---|
| *Mean NN* | -0.10; 0.003 | -0.39; <0.001 | -0.36; <0.001 | -0.26; 0.03 | -0.33; <0.001 | -0.16; 0.009 |
| *SDNN* | 0.14; 0.14 | 0.04; 0.40 | 0.06; 0.36 | 0.02; 0.44 | -0.11; 0.13 | -0.07; 0.05 |
| *RMSSD* | 0.15; 0.11 | 0.10; 0.81 | 0.25; 0.81 | -0.02; 0.47 | -0.09; 0.22 | -0.12; 0.01 |
| *pNN50* | 0.24; 0.21 | 0.13; 0.90 | 0.17; 0.85 | 0.00; 0.48 | -0.12; 0.21 | -0.10; 0.03 |
| *HF* | 0.17; 0.24 | 0.12; 0.86 | 0.18; 0.89 | 0.02; 0.75 | -0.06; 0.40 | -0.14; 0.03 |
| *LF* | 0.22; 0.21 | 0.12; 0.67 | 0.26; 0.62 | -0.03; 0.39 | -0.11; 0.16 | -0.12; 0.03 |

| | | | | | | |
|---|---|---|---|---|---|---|
| *LF/HF* | 0.19; 0.50 | -0.17; 0.60 | -0.20; 0.56 | 0.00; 0.82 | -0.14; 0.78 | -0.10; 0.32 |
| *Sample Entropy* | 0.19; 0.87 | 0.00; 0.99 | 0.23; 0.77 | -0.13; 0.30 | -0.16; 0.23 | -0.09; 0.24 |

**Table S19.** Comparison of correlations between HRV analysis parameters and clinical measures in a group of patients with heart failure. The values are represented as a correlation coefficient; p-value. The correlation was determined using the Pearson method for normal distributions of both variables or the Spearman method when the assumption of normal distributions was not fulfilled.

| | *Length of hospitalization (≤8 or >8 days)* | *NIHSS discharge (≥9 or <9)* | *mRS discharge (≥3 or <3)* | *Death* | *Nosocomial pneumonia* | *Nosocomial urinary tract infection* |
|---|---|---|---|---|---|---|
| *Mean NN* | -0.77; 0.006 | -0.29; 0.96 | -0.37; 0.006 | -0.40; 0.49 | 0.00; 0.12 | - |
| *SDNN* | -0.54; 0.05 | 0.12; 0.46 | 0.07; 0.30 | 0.10; 0.87 | -0.30; 0.16 | - |
| *RMSSD* | -0.11; 0.67 | 0.12; 0.31 | 0.12; 0.74 | -0.20; 0.17 | -0.36; 0.005 | - |
| *pNN50* | 0.22; 0.89 | 0.12; 0.16 | 0.18; 0.55 | -0.30; 0.06 | -0.42; <0.001 | - |
| *HF* | -0.21; 0.67 | 0.12; 0.31 | 0.17; 0.74 | -0.20; 0.17 | -0.36; 0.005 | - |
| *LF* | -0.84; 0.02 | 0.23; 0.75 | -0.11; 0.26 | 0.10; 0.90 | -0.18; 0.34 | - |
| *LF/HF* | -0.91; 0.006 | 0.23; 0.27 | -0.12; 0.04 | 0.50; 0.003 | 0.48; 0.01 | - |
| *Sample Entropy* | 0.64; 0.002 | -0.12; 0.60 | 0.34; 0.006 | -0.40; 0.05 | -0.48; 0.02 | - |

*Too small a group of patients with heart failure and nosocomial urinary tract infection to make a correlation.

**Table S20.** Comparison of correlations between HRV analysis parameters and clinical measures in a group of patients without heart failure. The values are represented as a correlation coefficient; p-value. The correlation was determined using the Pearson method for normal distributions of both variables or the Spearman method when the assumption of normal distributions was not fulfilled.

| | *Length of hospitalization (≤8 or >8 days)* | *NIHSS discharge (≥9 or <9)* | *mRS discharge (≥3 or <3)* | *Death* | *Nosocomial pneumonia* | *Nosocomial urinary tract infection* |
|---|---|---|---|---|---|---|
| *Mean NN* | -0.09; 0.006 | -0.34; 0.001 | -0.34; <0.001 | -0.22; 0.002 | -0.33; <0.001 | -0.22; 0.002 |

| | | | | | | |
|---|---|---|---|---|---|---|
| *SDNN* | 0.13; 0.02 | 0.05; 0.65 | 0.11; 0.58 | -0.05; 0.52 | -0.03; 0.26 | -0.16; 0.01 |
| *RMSSD* | 0.12; 0.007 | 0.14; 0.97 | 0.33; 0.86 | -0.01; 0.89 | 0.02; 0.60 | -0.19; 0.005 |
| *pNN50* | 0.20; 0.02 | 0.16; 0.98 | 0.21; 0.88 | 0.01; 0.80 | 0.03; 0.52 | -0.16; 0.01 |
| *HF* | 0.15; 0.03 | 0.16; 0.73 | 0.35; 0.82 | 0.02; 0.82 | 0.03; 0.86 | -0.20; 0.01 |
| *LF* | 0.22; 0.03 | 0.11; 0.88 | 0.30; 0.82 | -0.13; 0.67 | -0.06; 0.38 | -0.19; 0.01 |
| *LF/HF* | 0.22; 0.14 | -0.25; 0.36 | 0.16; 0.55 | -0.15; 0.31 | -0.32; 0.47 | -0.14; 0.17 |
| *Sample Entropy* | 0.11; 0.25 | 0.09; 0.59 | 0.25; 0.37 | -0.04; 0.69 | -0.02; 0.98 | -0.08; 0.22 |

**Table S21.** Comparison of correlations between HRV analysis parameters and clinical measures in a group of patients with diabetes. The values are represented as a correlation coefficient; p-value. The correlation was determined using the Pearson method for normal distributions of both variables or the Spearman method when the assumption of normal distributions was not fulfilled.

| | *Length of hospitalization (≤8 or >8 days)* | *NIHSS discharge (≥9 or <9)* | *mRS discharge (≥3 or <3)* | *Death* | *Nosocomial pneumonia* | *Nosocomial urinary tract infection* |
|---|---|---|---|---|---|---|
| *Mean NN* | 0.82; 0.08 | 0.00; 0.30 | 0.07; 0.47 | -0.41; 0.12 | 0.04; 0.21 | 0.17; 0.30 |
| *SDNN* | -0.15; 0.02 | 0.43; 0.04 | 0.50; 0.06 | 0.06; 0.60 | 0.65; 0.004 | -0.52; <0.001 |
| *RMSSD* | -0.14; 0.02 | 0.43; 0.04 | 0.50; 0.06 | 0.06; 0.60 | 0.65; 0.004 | -0.52; <0.001 |
| *pNN50* | -0.25; 0.02 | 0.50; 0.03 | 0.57; 0.04 | 0.17; 0.51 | 0.72; 0.002 | -0.52; <0.001 |
| *HF* | -0.10; 0.02 | 0.43; 0.04 | 0.50; 0.06 | 0.06; 0.60 | 0.65; 0.004 | -0.52; <0.001 |
| *LF* | -0.07; 0.02 | 0.50; 0.02 | 0.50; 0.07 | 0.06; 0.62 | 0.65; 0.003 | -0.41; <0.001 |
| *LF/HF* | 0.19; 0.01 | 0.14; 0.49 | -0.21; 0.18 | -0.52; 0.10 | -0.19; 0.26 | 0.41; 0.008 |

| | | | | | | |
|---|---|---|---|---|---|---|
| *Sample Entropy* | -0.24; 0.05 | 0.28; 0.07 | 0.64; 0.002 | 0.29; 0.19 | 0.42; 0.09 | -0.41; <0.001 |

**Table S22.** Comparison of correlations between HRV analysis parameters and clinical measures in a group of patients without diabetes. The values are represented as a correlation coefficient; p-value. The correlation was determined using the Pearson method for normal distributions of both variables or the Spearman method when the assumption of normal distributions was not fulfilled.

| | *Length of hospitalization (≤8 or >8 days)* | *NIHSS discharge (≥9 or <9)* | *mRS discharge (≥3 or <3)* | *Death* | *Nosocomial pneumonia* | *Nosocomial urinary tract infection* |
|---|---|---|---|---|---|---|
| *Mean NN* | -0.23; <0.001 | -0.43; <0.001 | -0.42; <0.001 | -0.22; 0.06 | -0.39; <0.001 | -0.19; <0.001 |
| *SDNN* | 0.09; 0.25 | 0.02; 0.33 | 0.03; 0.37 | -0.02; 0.10 | -0.20; 0.007 | -0.08; 0.17 |
| *RMSSD* | 0.16; 0.28 | 0.11; 0.64 | 0.26; 0.63 | -0.11; 0.03 | -0.16; 0.06 | -0.11; 0.26 |
| *pNN50* | 0.30; 0.35 | 0.10; 0.54 | 0.12; 0.60 | -0.08; 0.04 | -0.17; 0.04 | -0.08; 0.34 |
| *HF* | 0.15; 0.51 | 0.15; 0.99 | 0.28; 0.97 | -0.09; 0.11 | -0.14; 0.15 | -0.13; 0.33 |
| *LF* | 0.04; 0.40 | 0.11; 0.61 | 0.15; 0.64 | -0.08; 0.07 | -0.21; 0.02 | -0.13; 0.23 |
| *LF/HF* | 0.14; 0.94 | -0.23; 0.48 | -0.20; 0.55 | 0.13; 0.17 | -0.17; 0.91 | -0.21; 0.72 |
| *Sample Entropy* | 0.26; 0.68 | 0.04; 0.96 | 0.19; 0.65 | -0.21; 0.05 | -0.17; 0.24 | -0.05; 0.91 |

**Figure S1.** A scatter plot of the relationship between two variables. People with a favorable stroke outcome were marked in green, and people with an unfavorable stroke outcome in red. The plot was made using the pairplot function from the seaborn package (Python). The line shows the trend in the data (kind="reg"). The X- and Y-axes contain, respectively: Mean NN, SDNN, RMSSD, pNN_50, HF, LF, LF_HF_ratio, sampEnt, NIHSS at discharge >=9 or <9, hospitalization > 8 days or <=8 days, mRS at discharge >=3 or <3, death. Correlation coefficients and p-values of individual pairs of variables are included in the main text in Table 4.

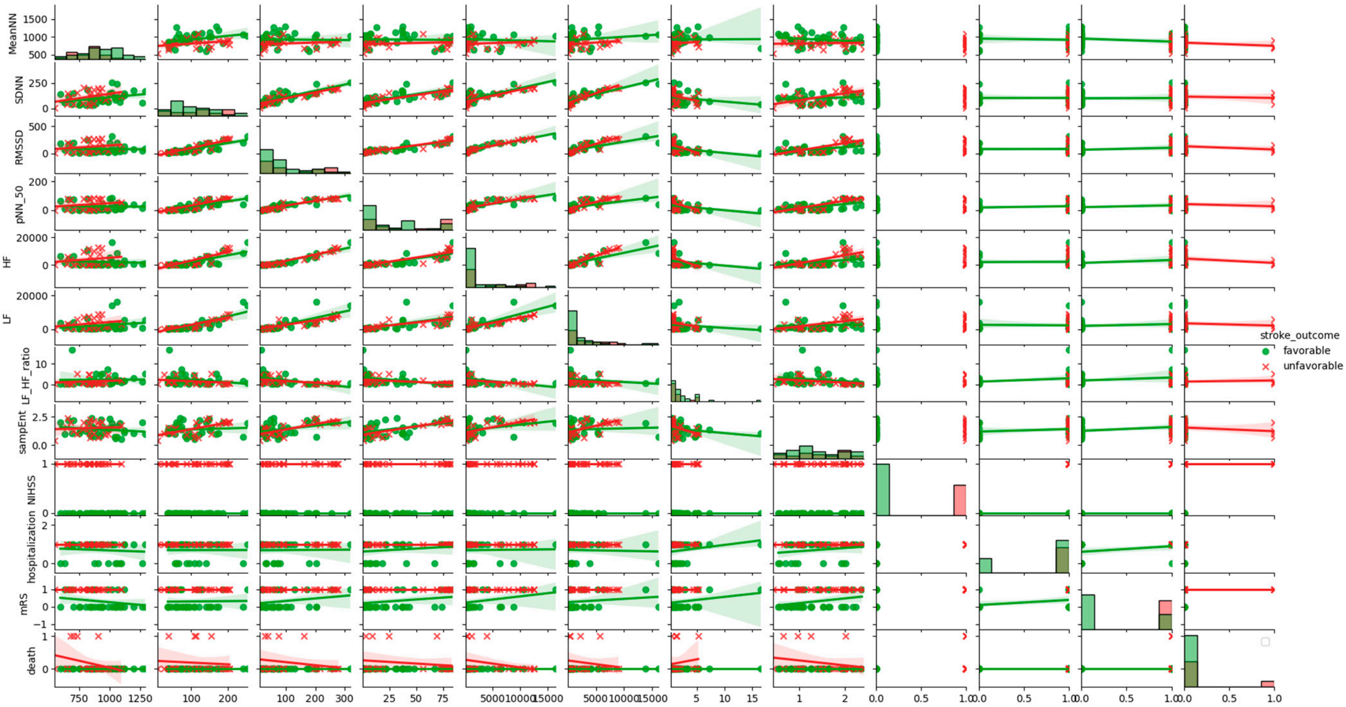
MeanNN
SDNN
RMSSD
pNN_50
HF
LF
LF_HF_ratio
sampEnt
NIHSS
hospitalization
mRS
death
stroke_outcome
favorable
unfavorable
